# Role of liver-resident NK cells in liver immunity

**DOI:** 10.1007/s12072-025-10778-7

**Published:** 2025-02-01

**Authors:** Zheng Pan, Yan-shuo Ye, Chang Liu, Wei Li

**Affiliations:** Department of Hepatobiliary-Pancreatic Surgery, China-Japan Union Hospital of Jilin University, Changchun, 130033 China

**Keywords:** Liver, Immunity, Natural killer (NK) cells, Liver-resident natural killer (LrNK) cells

## Abstract

The tolerogenic immune microenvironment of the liver (the immune system avoids attacking harmless antigens, such as antigens derived from food and gut microbiota) has garnered significant attention in recent years. Inherent immune cells in the liver play a unique role in regulating this microenvironment. Liver-resident natural killer (LrNK) cells, also known as liver type 1 innate lymphoid cells (ILC1s), are a recently discovered subset of immune cells that possess properties distinct from those of conventional NK (cNK) cells. Accumulating evidence suggests that there are significant differences between LrNK and cNK cells, with LrNK cells potentially exhibiting immunosuppressive functions in the liver. This review summarizes the latest findings on LrNK cells, focusing on their phenotype, heterogeneity, plasticity, origin, development, and the required transcription factors. In addition, immune functions of LrNK cells in various liver diseases, including liver cancer, viral infections, liver injury, and cirrhosis, were analyzed. By elucidating the role of LrNK cells in liver immunity, this review aims to enhance our understanding of the mechanisms underlying liver immunity and contribute to the improvement of liver disease treatment.

## Introduction

The liver has long been recognized as an immunologically privileged organ characterized by a microenvironment that tends to be more tolerogenic than immunogenic [1]. Despite the constant influx of foreign antigens from food and toxins secreted by intestinal symbiotic bacteria through the portal vein, the liver typically does not develop immune responses under stable conditions [2, 3]. This unique property enables certain hepatotropic viruses, such as hepatitis B virus (HBV) and hepatitis C virus (HCV), to evade immune clearance and establish chronic infections [4, 5]. Remarkably, liver allografts exhibit exceptional tolerance in various animal models. Spontaneous acceptance without immunosuppressive drugs has been observed in mice, some rat strains and pigs [6–8]. In humans, liver allografts demonstrate significantly less severe immune rejection than kidney, heart, and lung transplants [9]. Intriguingly, a phenomenon known as “operational tolerance” occurs in some liver transplant recipients, where complete withdrawal of immunosuppressive drugs is possible without subsequent immune rejection [10, 11].

However, the mechanisms underlying liver tolerance remain largely unknown. Accumulating evidence suggests that various liver non-parenchymal cells (NPCs) play crucial roles in inducing this tolerogenic state. These include hepatic stellate cells (HSCs), liver sinusoidal endothelial cells (LSECs), Kupffer cells (KCs), dendritic cells (DCs), regulatory T (Treg) cells, B cells, natural killer (NK) cells, and natural killer T (NKT) cells [12–15].

Recently, a novel population of liver-resident NK (LrNK) cells, also known as liver type 1 innate lymphocytes (ILC1s), has been discovered in a mouse model of hapten-induced contact hypersensitivity (CHS), which endows this hypersensitivity response [16]. These cells share some similarities with circulating conventional NK (cNK) cells, as they express both NK1.1^+^ and natural killer p46^+^ (NKp46^+^). However, LrNK cells are distinguished by their persistent residence in the liver and lack of circulation in the peripheral blood [16]. Recent studies have revealed significant differences between LrNK and cNK cells in terms of their origin, developmental pathways, phenotypes, and immune functions [17]. These distinctions suggest that LrNK cells may play a unique role in shaping the fascinating microenvironment of the liver and potentially contribute to the induction of tolerance in liver transplantation. The advent of advanced technologies, particularly single-cell RNA sequencing (scRNA-Seq), has revolutionized our ability to study specific cell populations. This technique allows the analysis of individual cell transcriptomes, providing a more precise understanding of cellular heterogeneity than bulk transcriptome data. The application of scRNA-Seq in liver immunology has the potential to unravel the complexities of LrNK cells and their roles in liver immunity. In this review, we summarize the latest findings on LrNK cells and highlight the potential directions for future research in this field. By synthesizing the current knowledge and identifying gaps in our understanding, we aimed to provide a comprehensive overview of LrNK cells and their potential role in liver immunity.

## Physiology of LrNK cells

### The phenotype of LrNK cells

Phenotypically, mouse LrNK cells are universally recognized as CD49a^+^ CD49b^−^ (DX5^−^) Lin^−^, whereas cNK cells are recognized as CD49a^−^ CD49b^+^ Lin^−^. LrNK cells co-express NK1.1, NKp46, C-X-C motif chemokine receptor 6 (CXCR6), tumor necrosis factor (TNF)-related apoptosis-inducing ligand (TRAIL), and CD69 on their cell membrane surface [16–19]. As research on LrNK cells has progressed, our understanding of how to distinguish LrNK cells from cNK cells has deepened. Recent studies have revealed more nuanced approaches to identifying and characterizing LrNK cells. For example, Lin cocktails vary among studies and are susceptible to contamination by Lin^+^ cells, especially T cells. Therefore, the combination of CD3ϵ/T cell receptor^−^ (TCR^−^) markers may minimize T cell contamination and improve specificity when identifying LrNK cells [20]. Mouse multi-tissue scRNA-Seq and cellular indexing of transcriptomes and epitopes by sequencing (CITE-Seq) studies have identified syndecan-4 as a potential biomarker for distinguishing cNK from tissue-resident NK cells, including those in the liver [21]. However, CD49a remains the core marker of LrNK cells, with syndecan-4 serving as an auxiliary marker under certain conditions. NKp46 (a cell surface protein on LrNK cells) controls the expression of TRAIL (another cell surface protein on LrNK cells) at the post-translational level. Specifically, NKp46 may facilitate TRAIL protein trafficking to the cell membrane, ensuring TRAIL-dependent cytotoxicity [22–24]. The interactions between different receptors or effector molecules in mouse LrNK cells warrant further investigation to uncover potential biological insights.

Human LrNK cell identification remains controversial, with different research groups proposing various panels based on their methodology [25]. A recent study using 29-color panel flow cytometry to investigate bulk human liver NK cells, including cNK and LrNK cells, suggested that CD49e^−^ could serve as a phenotypic marker for human LrNK cells [26]. However, this finding required further validation. Given the important roles of mouse LrNK cells in various liver diseases (described below), it is speculated that human LrNK cells may play similar roles in human liver pathologies. To advance our understanding of human LrNK cells, further research utilizing multi-omics technologies, such as scRNA-Seq, CITE-Seq, and spatial omics, is crucial. These approaches will help to identify the characteristics of human LrNK cells and elucidate their biological functions, potentially contributing to the development of novel treatments for liver diseases.

### Heterogeneity and plasticity of LrNK cells

The heterogeneity of LrNK cells remains subject of debate in the scientific community. While one study suggested the uniqueness of LrNK cells, identifying CD49a^+^ as a reliable marker to distinguish them from cNK cells using scRNA-seq and metacell modeling [27], other studies have demonstrated the heterogeneity of mouse LrNK cells [21, 28, 29]. One study used a combination of CD160 and Granzyme A (GzmA) to further characterize mouse LrNK cells. CD160^+^ LrNK cells exhibit lower cytotoxicity, whereas GzmA^+^ LrNK cells have a lower capacity to produce interferon-γ (IFN-γ) [28]. Another study found that LrNK cells could be further divided into Ly49E^+^ and Ly49E^−^ LrNK cells. Ly49E^+^ LrNK cells show cytotoxicity against murine cytomegalovirus (MCMV) infection in newborn mice, while Ly49E^−^ LrNK cells exhibit immune memory in the classical hapten-induced CHS model [29]. Furthermore, Lopes et al. identified two distinct subpopulations of mouse LrNK cells, LrNK Liv1 and LrNK Liv2 [21]. LrNK Liv1 cells preferentially express *Il7r*, *Tmem176a*, *Tmem176b*, *Ikzf2*, and *Cd160*, which are associated with cell adhesion, IFN-γ production, and defense responses against bacteria. In contrast, LrNK Liv2 cells preferentially express *Itga1*, *Cd3g*, *Cd7*, and *Gzmc* genes, which are associated with cytokine production and antigen receptor-mediated signaling pathways [21]. The discrepancies among these studies may be attributed to variations in sample processing procedures and analysis methods (Table 1). Moreover, from a technical perspective, it is important to note that unsupervised clustering techniques can divide a cell population into any multiple subpopulations. However, the biological significance of these subpopulations requires further validation using in vivo and in vitro experiments. Further studies are warranted to determine whether these identified subpopulations represent different states of the same cell type and whether there is an overlap between subpopulations.

**Table 1:** The different sample processing procedures and analysis methods used in the studies

Reference	Sample processing procedures	Analysis methods
[27]	70 mM filter, and 30%, 70% Percoll density gradient centrifugation	Massively parallel single-cell RNA-seq and metacell modeling
[21]	100 mm-mesh cell strainers, and 37.5%, 67.5% Percoll density gradient centrifugation	Cellular indexing of transcriptomes and epitopes by sequencing and Seurat (v3.1.5)
[28]	70 μm cell strainer, 38.5% Percoll centrifuging at 325×*g* for 20 min	Single-cell RNA-seq analysis and Seurat (v4.0.1) aiming NK1.1^+^ NKp46^+^ cells
[29]	200-gauge mesh, and 40%, 70% Percoll density gradient centrifugation	Single-cell RNA-seq analysis and Seurat (v3.0) aiming CD45^+^ NK1.1^+^ NKp46^+^ CD3^−^ CD19^−^ cells

*NKp*46 natural killer p46

Recent studies have reported the plasticity between cNK and LrNK cells. The expression of liver-homing receptors CXCR6 and CD69 in human peripheral blood CD56^bright^ NK cells may contribute to their liver residence [30]. In addition, stimulation with interleukin-12 (IL-12) and IL-15 can induce the conversion of human peripheral blood NK cells into CD49a^+^ CXCR6^+^ NK cells with a CD56^bright^ CD69^+^ NKG2C^+^ phenotype, which is similar to that of human LrNK cells [31]. Further research is needed to elucidate whether the conversion between cNK and LrNK cells occurs routinely under physiological homeostasis or only in response to certain stimuli and the key factors influencing this conversion process.

### Origin, development, and transcription factors of LrNK cells

Our understanding of the origin and development of LrNK cells remains limited, although a recent study has elucidated these aspects. A previous study identified a small population of Lin^−^ Sca-1^+^ Mac-1^+^ (LSM) hematopoietic stem cells in the mouse liver, capable of differentiating into Lin^−^ CD122^+^ CD49a^+^ cells and subsequently into LrNK cells [32]. Interestingly, mouse LrNK cells secrete IFN-γ, creating a positive feedback loop that promotes the expansion and differentiation of LSM hematopoietic stem cells into LrNK cells [32]. This study also revealed that mouse LrNK cells are produced through extramedullary hematopoiesis, specifically in the liver. Further evidence supporting this localized development came from a study demonstrating that liver irradiation impeded mouse LrNK cell development, even when precursor cells from the bone marrow, liver, and spleen were transferred [33].

Cell–cell contact interactions play a crucial role in the development and maturation of LrNK cells. For instance, CD8^+^ T cells have been shown to promote the functional maturation of mouse LrNK cells via the CD70-CD27 axis [34]. Moreover, parenchymal cells such as hepatocytes contribute to the development and maintenance of mouse LrNK cells by secreting IL-15 [35]. Interestingly, factors beyond the liver also influence the development of LrNK cells. Early life butyric acid, produced by intestinal microbiota, stimulates Kupffer cells and hepatocytes to secrete IL-18, which maintains mitochondrial activity and promotes the maturation of mouse LrNK cells [36]. These findings highlight the complex interplay between intrahepatic and extrahepatic factors in LrNK cells development, maturation, and maintenance through diverse mechanisms.

Several transcription factors are involved in LrNK cells development and function. These include the well-established T-box expressed in T cells (T-bet), homolog of blimp-1 in T cells (Hobit), and the aryl hydrocarbon receptor (AhR) [37–40]. Recently, a novel transcription factor, RAR-related orphan receptor alpha (RORα), was found to play a role in the development and anti-tumor function of mouse LrNK cells [41]. The existence of other transcription factors requires further investigation.

### LrNK cells in other species

Researchers have gradually identified and characterized LrNK cells in species other than the extensively studied LrNK cells in mice and humans. In porcine models, CD8α^dim^ CD3^−^ cells have been recognized as LrNK cells, exhibiting a distinct Eomes^high^ T-bet^low^ CXCR6^+^ CD49e^−^ phenotype [42]. Consistent with LrNK cells in other species, porcine LrNK cells demonstrate high expression of adaptive immune response-related genes and low expression of cellular migration-related genes, indicating an immature anti-inflammatory phenotype [43]. Porcine LrNK cells differ from their counterparts in other species in terms of their cytotoxic capacity. Unlike LrNK cells in mice and humans, porcine LrNK cells do not exhibit cytotoxic activity against K562 cells (a human immortalized myelogenous leukemia cell line) or pseudorabies virus (PRV)-infected cells. This unique characteristic warrants further investigation to elucidate the functional implications of the difference. Lin, et al. conducted a cross-species comparison and found that porcine LrNK cells exhibit a transcription factor expression pattern more closely resembling that of human LrNK cells than mouse LrNK cells. Specifically, both human and porcine LrNK cells express high levels of *EOMES* and low levels of T-Box transcription factor 21 (*TBX21*), whereas mouse LrNK cells display the opposite pattern with high *TBX21* and low *EOMES* expression [44]. These findings suggest that porcine models may offer a more suitable platform for studying LrNK cells characteristics and functions in health and disease, potentially providing more translatable insights into human biology than mouse models.

## Functions of LrNK cells

### LrNK cells in liver cancer

LrNK cells appear to play a crucial immunoregulatory role in liver cancer. Recent studies have shed light on their potential involvement in tumor progression and prognosis, particularly in hepatocellular carcinoma (HCC). A pivotal study demonstrated that human CD49a^+^ LrNK cells exhibit a regulatory phenotype and accumulated in HCC tissues. Notably, the presence of these cells correlates with tumor progression and poor prognosis [45], suggesting a tumor-promoting role for CD49a^+^ human LrNK cells [45]. This finding challenges the conventional understanding of NK cells as primary anti-tumor agents and highlights the complex nature of tumor immunology. Furthermore, in a murine model, Tim-3, an inhibitory receptor, was found to be upregulated in tumor-infiltrating LrNK cells, which could dampen the anti-tumor effect of LrNK cells by inhibiting the phosphatidylin-ositol-3-kinase (PI3K)/Akt/mammalian target of rapamycin (mTOR) signaling pathway [46]. The PI3K/Akt/mTOR pathway is known to play a critical role in cell proliferation, survival, and metabolism, suggesting that its inhibition in LrNK cells could significantly impair their anti-tumor functions.

Collectively, these findings suggest that specifically targeting the immunoregulatory function of LrNK cells could represent a novel therapeutic approach for HCC. By modulating the activity of these cells, it may be possible to enhance the overall anti-tumor immune response and improve patient outcomes. However, this conclusion requires further validation using both in vivo and in vitro experiments.

### LrNK cells in viral infection

**Fig. 1:**
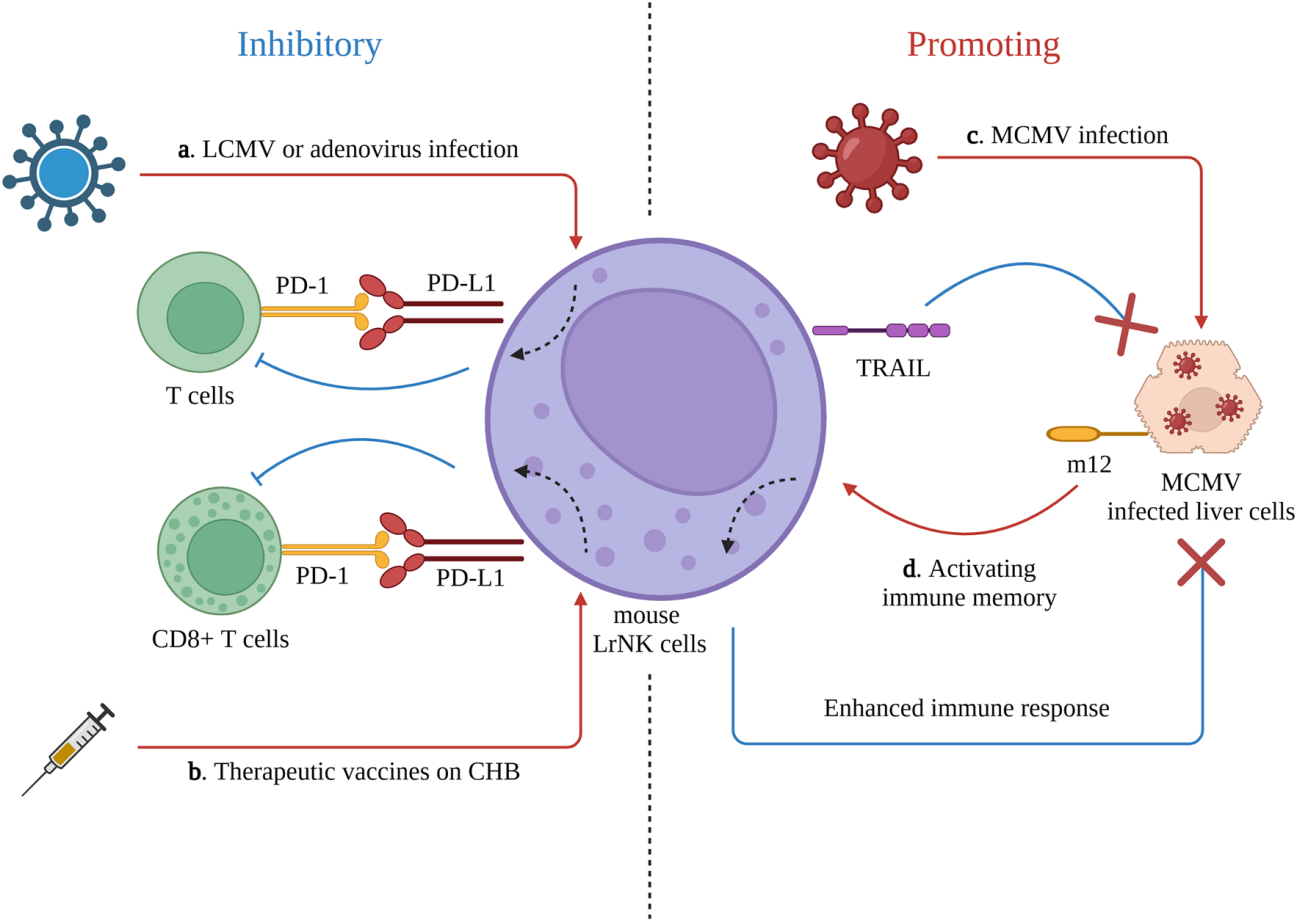
The role of mouse LrNK cells in hepatic viral infection. **a.** In LCMV or adenovirus infection, T cells were inhibited by mouse LrNK cells via the PD-1 and PD-L1 axis. **b.** After the therapeutic vaccinating on CHB, the CD8^+^ T cells were also inhibited by mouse LrNK cells via the same PD-1 and PD-L1 axis. **c.** In MCMV infection, mouse LrNK cells could kill the infected cells by TRAIL, **d.** meanwhile the viral glycoprotein, m12, conferred the immune memory of mouse LrNK cells. Abbreviations: *LrNK cells* liver-resident natural killer cells, *LCMV* lymphocytic choriomeningitis, *PD-*1 programmed cell death protein 1, *PD-L*1 programmed cell death ligand 1, *CHB* chronic hepatitis B, *MCMV* murine cytomegalovirus, *TRAIL* tumor necrosis factor (TNF)-related apoptosis-inducing ligand

Notably, LrNK cells appear to negatively regulate adaptive immune responses in certain viral infections. In mouse models of lymphocytic choriomeningitis virus (LCMV) and adenovirus infection, LrNK cells inhibited the antiviral activity of virus-specific T cells through the programmed cell death protein 1 (PD-1) and programmed cell death ligand 1 (PD-L1) axes [47] (Fig. 1a). Similarly, intrahepatic HBV-specific CD8^+^ T cells induced by therapeutic vaccine injection were suppressed by LrNK cells through the same PD-1 and PD-L1 axes in mice [48] (Fig. 1b). In patients with HCV infection, CD56^Bright^ CD16^−^ human LrNK cells are associated with improved liver function (e.g., attenuated immune-mediated liver injury) [49]. This observation confirms the immunoregulatory role of LrNK cells in specific types of hepatotropic and immunologically resistant viral infections (Fig. 1-a, b). Targeting LrNK cells may potentially eliminate the immune tolerance and chronic infections caused by these viruses.

In contrast, LrNK cells play different roles during CMV infection. Studies have demonstrated that murine LrNK cells can eliminate MCMV-infected cells through the TRAIL protein, which binds to its receptor, activates the caspase cascade, and induces apoptosis. In addition, the viral glycoprotein m12 on the surface of MCMV-infected cells can stimulate LrNK cells to form an immune memory [50, 51] (Fig. 1c, d). Consistent with these findings, a subpopulation of human LrNK cells, CD2^+^ LrNK cells, expanded and exhibited antiviral activity during human CMV infection [52]. This divergent response may be related to the specificity of the CMV itself, such as the m12 protein mentioned above, which warrants further investigation.

### LrNK cells in liver injury

**Fig. 2:**
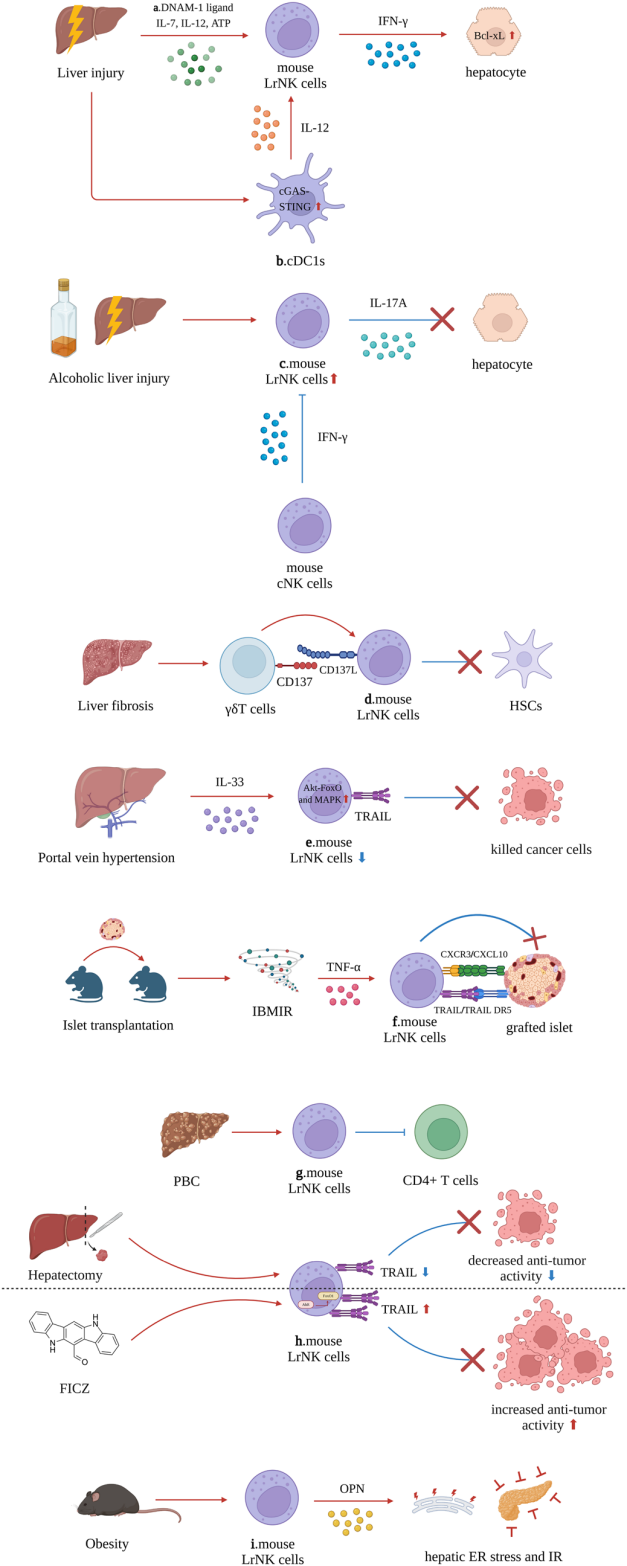
The role of mouse LrNK cells in other liver diseases (except for liver viral infection and liver cancer). **a.** In liver injury, the release and increase of DNAM-1 ligand, IL-7, IL-12, ATP could stimulate mouse LrNK cells to secrete IFN-γ, which protected hepatocytes from apoptosis by upregulating Bcl-xL. **b.** And the cGAS-STING signaling pathway within cDC1s was also activated and cDC1s secrete IL-12, which could promote the secretion of IFN-γ by mouse LrNK cells. **c.** In alcoholic liver injury, mouse LrNK cells were stimulated to secrete IL-17A and exacerbated liver injury. While the cNK cells could secrete IFN-γ, which inhibited the activation of mouse LrNK cells. **d.** In liver fibrosis, γδT cells could activate mouse LrNK cells via the CD137L-CD137 axis to kill HSCs. So, the liver fibrosis improved. **e.** In portal vein hypertension, increased IL-33 could activate the Akt-FoxO and MAPK signaling pathways within mouse LrNK cells and decreased the expression of TRAIL on mouse LrNK cells. Thereby, the anti-tumor activity of mouse LrNK cells was diminished. **f.** In the mouse model of islet transplantation, the IBMIR-induced TNF-α could activate mouse LrNK cells to kill grafted islet by CXCR3-CXCL10 and TRAIL-TRAIL DR5 axes. **g.** In PBC, the CD4^+^ T cells were inhibited by mouse LrNK cells, which improve the immune injury. **h.** Partial hepatectomy could decrease the expression of TRAIL on mouse LrNK cells and attenuate the anti-tumor activity. Meanwhile, FICZ, the AhR agonist, could activate the AhR-FoxO1 within mouse LrNK cells, increased the expression of TRAIL and anti-tumor activity. **i.** In obesity, the mouse LrNK cells could secrete OPN, which promoted hepatic ER stress and IR. Abbreviations: *LrNK cells* liver-resident natural killer cells, *DNAM*-1 DNAX accessory molecule-1, *IL* interleukin, *ATP* adenosine triphosphate, *IFN-γ* interferon-γ, *cGAS* cyclic GMP-AMP, STING stimulator of interferon genes, *cDC1s* type 1 conventional dendritic cells, *cNK* conventional natural killer cells, *HSCs* hepatic stellate cells, *TRAIL* tumor necrosis factor (TNF)-related apoptosis-inducing ligand, *IBMIR* instant blood-mediated inflammatory reaction, *TNF-α* tumor necrosis factor α, *TRAIL DR*5 TRAIL death receptor 5, *PBC* primary biliary cholangitis, *FICZ* 6-formylindolo[3,2-b]carbazole, *AhR* aryl hydrocarbon receptor, *OPN* osteopontin, *ER* endoplasmic reticulum, *IR* insulin resistance

Studies have demonstrated that LrNK cells play diverse roles in various types of liver injuries in mice. Depending on the specific context of the liver damage, these cells exhibit both protective and detrimental effects. In CCl_4_-induced liver damage, increased levels of DNAM-1 ligand, IL-7, IL-12, and adenosine triphosphate (ATP) stimulate mouse LrNK cells to secrete IFN-γ and protect liver cells from further damage by upregulating the endogenous anti-apoptotic protein Bcl-xL [53] (Fig. 2a). The cyclic GMP-AMP synthase (cGAS) and cyclic GMP-AMP receptor stimulator of interferon genes (STING) (cGAS-STING) signaling pathways are activated in the mouse conventional type 1 dendritic cells (cDC1s). This activation facilitates IL-12 secretion, which further stimulates IL-18r^+^ LrNK cells to secrete IFN-γ, thereby enhancing the protective effect [54] (Fig. 2b).

Conversely, LrNK cells appear to play a detrimental role in certain types of liver injuries. In a mouse model of steatotic liver ischemia–reperfusion injury, the number of LrNK cells increased and exhibited a pro-inflammatory phenotype, exacerbating liver injury [55]. In a mouse model of alcoholic liver injury, the proportion of LrNK cells increased boosting liver injury through IL-17A secretion [56]. Intriguingly, cNK cells secrete IFN-γ, which blocks IL-17A secretion by LrNK cells, potentially attenuating liver injury [56] (Fig. 2c).

Therefore, LrNK cells exhibit context-dependent activation patterns, depending on the type of liver injury. This variability results in either a promotion or protective role in liver damage. Further studies are necessary to elucidate the specific activation mechanisms of LrNK cells in different types of liver injuries. Understanding these mechanisms could potentially allow for harnessing of their potential protective effects against liver damage.

### LrNK cells in cirrhosis progression

Recent studies have highlighted the potential role of LrNK cells in ameliorating liver fibrosis. In a CCl_4_-induced mouse liver fibrosis model, liver γδT cells were found to enhance the cytotoxicity of LrNK toward HSCs via the CD137 and CD137L axes [57] (Fig. 2d). This finding suggests that targeting the interaction between LrNK cells and HSCs may be a promising therapeutic approach for liver fibrosis. Furthermore, research has demonstrated that activating LrNK cells to target HSCs may yield antifibrotic effects. Two recent studies have found that testosterone [58] and *Ecballium elaterium* (a plant commonly used in Mediterranean medicine) [59] showed antifibrotic effects through mouse LrNK cells activation.

A subpopulation of human LrNK cells, termed “liver-type” LrNK cells, characterized by the CD49a^+^ CD94^+^ CD200R1^+^ phenotype, has been recently identified through a combination of proteomics and scRNA-Seq techniques [60]. Notably, these “liver-type” LrNK cells exhibited extensive proliferation in cirrhotic liver tissue. Functionally, “liver-type” LrNK cells can produce various cytokines, including, IL-2, IFN-γ, TNF-α, and granulocyte–macrophage colony-stimulating factor (GM-CSF), but lack the cytotoxic molecules perforin and granzyme B [60]. This phenomenon is interesting. Although these findings are compelling, the precise role of “liver-type” LrNK cells in the initiation and progression of liver cirrhosis remains to be elucidated and warrants further investigation. Portal hypertension, the most common and serious complication of liver cirrhosis, has been linked to alterations in LrNK cell function. In a mouse model of portal hypertension, elevated concentrations of IL-33 activated Akt-FoxO and mitogen-activated protein kinase (MAPK) signaling pathways in LrNK cells. This activation led to a reduction in the number of TRAIL^+^ LrNK cells and attenuated their anti-tumor activity [61] (Fig. 2e).

Therefore, LrNK cells appear to play multifaceted roles throughout the progression of liver cirrhosis, from the initial stages of fibrosis and cirrhosis to the development of complications, such as portal hypertension. Further studies on their functions at different stages of liver cirrhosis will undoubtedly enhance our understanding of the disease process and may lead to the development of novel therapeutic strategies. Elucidating the complex interplay between LrNK cells and other cellular components of the liver microenvironment will be crucial for the effective treatment and management of liver cirrhosis.

### LrNK cells in other diseases

The role of LrNK cells varies across diseases, and the underlying mechanisms remain largely unknown. Initially recognized in hapten-induced CHS, the processes of sensitization, immune memory formation, and liver retention of LrNK cells are still not fully understood. One study reported that, after initial hapten stimulation, IL-7Rα^+^ mouse “NK cells” are recruited to the draining lymph nodes of stimulated skin via CXCR3, where they gain immune memory [62]. Subsequently, these cells exit the lymph nodes and preferentially reside in the liver through CXCR6-mediated mechanisms [62]. Further research is needed to elucidate the behavior of LrNK cells following the second hapten stimulation.

LrNK cells play crucial roles in organ transplantation. In a mouse model of islet transplantation, TNF-α produced during the instant blood-mediated inflammatory reaction (IBMIR) activated LrNK cells, leading to islet grafts destruction through TRAIL-TRAIL death receptor 5 (DR5) and CXCR3-C-X-C motif chemokine ligand 10 (CXCL10) axes [63] (Fig. 2f). Conversely, in an innate immune-tolerated orthotopic liver transplantation mouse model, scRNA-Seq and cytometry by time-of-flight analysis revealed that donor-derived LrNK cells persisted and formed a chimeric state with recipient-derived LrNK cells during the stable phase of immune tolerance, despite the replacement of 96% of donor-derived immune cells with recipient-derived cells [64]. Further functional analyses demonstrated significant activation of LrNK cells in immune-mediation and tolerance induction during the acute phase, with overexpression of inhibitory receptor-encoding genes such as *Klrc1*, *Cd200r1*, *Cd96*, and *Lag3* [64]. Moreover, human LrNK cells isolated from the liver perfusate significantly induced the death of allogeneic T cells, particularly CD8^+^ T cells, in vitro [65]. In a mouse model of autoimmune primary biliary cholangitis (PBC), LrNK cells exhibited an inhibitory effect on activated CD4^+^ T cells and protected the liver from autoimmune inflammation [66] (Fig. 2g). Collectively, these findings suggest an immunosuppressive function of LrNK cells and their potential involvement in liver tolerance induction; however, further studies are required to confirm this conclusion.

Hepatectomy also affects LrNK cell function. Partial liver resection in mice significantly inhibited TRAIL expression in LrNK cells and attenuated their cytotoxicity against tumor cells. Conversely, the AhR agonist, 6-formylindolo[3,2-b]carbazole (FICZ), increased the proportion of TRAIL^+^ LrNK cells by upregulating FoxO1 expression, thereby enhancing local anti-tumor immune activity [67] (Fig. 2h). Therefore, these findings suggest that AhR agonists could potentially be used perioperatively in HCC patients undergoing liver resection to enhance the anti-tumor activity of LrNK cells and reduce recurrence risk.

LrNK cells have also been associated with metabolism-related diseases. In a mouse model of obesity, LrNK cells secreted osteopontin (OPN), a protein that induces hepatic endoplasmic reticulum (ER) stress and promotes insulin resistance (IR) [68] (Fig. 2i).

### Clinical insights

These studies suggest that activating LrNK cells may offer potential therapeutic approaches for certain liver diseases. For instance, in patients with liver fibrosis, LrNK cells can potentially ameliorate these conditions by targeting and eliminating HSCs [58, 59]. Moreover, the activation of LrNK cells in liver transplant recipients may contribute to inducing transplant tolerance [64, 65]. However, it is crucial to note that current research on human LrNK cells is limited. Most of our understanding of LrNK cells stems from murine models rather than from human studies. Consequently, the generalizability of these findings to human physiology and pathology requires further rigorous verification. Meanwhile, several critical questions warrant further investigation. Primarily, researchers need to elucidate methods for specifically modulating LrNK cell activity in vivo, without compromising the normal function of other immune cells. This targeted approach is essential for developing safe and effective therapeutic strategies.

## Conclusion and future research

The fascinating immune properties of the liver have attracted significant attention in recent years. Elucidating the intrinsic mechanisms underlying liver immunity could prove invaluable in addressing persistent challenges, such as chronic liver infection, autoimmune disorders, fibrosis, tumors, and transplantation tolerance. LrNK cells, a recently discovered and characterized subset of immune cells, have emerged as crucial players in liver immunology. Understanding the functions of LrNK cells is essential to gain deeper insights into the inherent immunological characteristics of the liver.

Compared with other hepatic immune cells, research on LrNK cells remains limited. The factors that regulate the immune function of these cells have not been fully elucidated. Recent advancements, such as the integration of human peripheral blood NK cells into a microfluidic perfused liver on-a-chip model, offer promising avenues for investigation [69]. Extending this approach to LrNK cells in a microfluidic perfused liver on-a-chip model holds promise for providing an in vivo-like, dynamic, and visually accessible platform for studying their interactions with other immune cells during the development and progression of liver disease. In addition, cutting-edge technologies such as in vivo time-lapse microscopy, spatial transcriptomics, spatial multi-omics, and genetic lineage tracing have contributed to a more comprehensive and in-depth understanding of LrNK cells. Identifying the key factors and signaling pathways that regulate LrNK cells and potentially manipulate these cells could pave the way for novel therapeutic strategies and drug development for various liver diseases. By harnessing the unique properties of LrNK cells, researchers may be able to modulate liver immunity in a targeted manner, potentially leading to more effective treatments for chronic liver diseases.
